# Construction and analysis of a comprehensive protein interaction network of HCV with its host *Homo sapiens*

**DOI:** 10.1186/s12879-019-4000-9

**Published:** 2019-04-30

**Authors:** Qurat ul Ain Farooq, Faisal F. Khan

**Affiliations:** 1 College of Life Sciences and Bio Engineering, Beijing University of Technology, Beijing, China; 2 Institute of Integrative Biosciences, CECOS University of IT and Emerging Sciences, Peshawar, Pakistan

**Keywords:** Hepatitis C virus, Protein-protein interactions, Protein interaction network, Cytoscape, KEGG, Gene ontology

## Abstract

**Background:**

Hepatitis C Virus is becoming a major health problem in Asia and across the globe since it is causing serious liver diseases including liver cirrhosis, chronic hepatitis and hepatocarcinoma (HCC). Protein interaction networks presents us innumerable novel insights into functional constitution of proteome and helps us finding potential candidates for targeting the drugs.

**Methods:**

Here we present a comprehensive protein interaction network of Hepatitis C Virus with its host, constructed by literature curated interactions. The network was constructed and explored using Cytoscape and the results were further analyzed using KEGG pathway, Gene Ontology enrichment analysis and MCODE.

**Results:**

We found 1325 interactions between 12 HCV proteins and 940 human genes, among which 21 were intraviral and 1304 were HCV-Human. By analyzing the network, we found potential human gene list with their number of interactions with HCV proteins. ANXA2 and NR4A1 were interacting with 6 HCV proteins while we found 11 human genes which were interacting with 5 HCV proteins. Furthermore, the enrichment analysis and Gene Ontology of the top genes to find the pathways and the biological processes enriched with those genes. Among the viral proteins, NS3 was interacting with most number of interactors followed by NS5A and so on. KEGG pathway analysis of three set of most HCV- associated human genes was performed to find out which gene products are involved in certain disease pathways. Top 5, 10 and 20 human genes with most interactions were analyzed which revealed some striking results among which the top 10 host genes came up to be significant because they were more related to Influenza A viral infection previously. This insight provides us with a clue that the set of genes are highly enriched in HCV but are not well studied in its infection pathway.

**Conclusions:**

We found out a group of proteins which were rich in HCV viral pathway but there were no drugs targeting them according to the drug repurposing hub. It can be concluded that the cluster we obtained from MCODE contains potential targets for HCV treatment and could be implemented for molecular docking and drug designing further by the scientists.

## Background

Hepatitis C Virus or HCV is a small, single-stranded, positive-sense RNA virus belonging to the genus Hepacivirus of Flaviviridae family, discovered in 1989 [1]. It is responsible for severe and chronic liver diseases [2–4] and affects 200 million people globally with 17 million cases in Pakistan alone [5, 6]. The HCV pathogen causes acute and persistent diseases such as liver cirrhosis [6], chronic hepatitis [2], and hepatocarcinoma [7]. HCV genome is 9.6 kb in size [8] and is translated into four structural and six non-structural (NS) proteins. The structural HCV proteins are Core, E1, E2 and p7 protein which splits structural proteins from non-structural proteins. The non-structural HCV proteins are NS2, NS3, NS4A, NS4B, NS5A and NS5B [2, 9, 10]. Another HCV protein named Frameshift protein or F protein was also discovered in the late 90’s [9].

Protein-protein interactions can be mapped into networks and we can gain valuable insights into functional constitution of proteome by analyzing different parameters of the network such as its size and complexity. PPI networks can help us investigate differences between the normal and diseased states. We can obtain fundamental knowledge about a disease by finding novel pathways through analysis of the PPI network, by investigating subnetworks and its potential hubs involved in causing that specific disease [11, 12].

Protein interactions are determined by different high throughput experimental and computational methods which yields different types of protein-protein interaction data. The high throughput experimental techniques identify the interactions either directly or infer them indirectly by different approaches [11, 13] including Yeast 2 Hybrid (Y2H) and Tandem affinity purification combined with mass spectrometry (TAPMS). Experimental methods for PPI detection have many limitations such as high false positive rate, high cost, time-consuming, and laborious. New computational methods are being practiced successfully to evaluate and analyze the interaction data generated by high-throughput experimental approaches, and to predict novel protein interactions by gaining insights from the already known interactions. Computational methods provide a quick and low-cost alternative to the traditional experimental techniques to predict PPIs. An important advantage of computational methods over experimental is that we can study proteins by mapping the pairwise associations into a comprehensive network according to their distinct functional level [11, 14].

The interactome map of HCV with human cellular proteins has been previously constructed by using the traditional yeast 2 hybrid screens, and also using the computational methods as well. Among the studies done on HCV protein-protein interactions, some are small scale studies experimentally-verifying a small number of PPIs, while others are large scale studies involving large number of PPIs either verified experimentally, or predicted computationally [7, 15, 16]. Although the Hepatitis C virus interactome has been constructed by many scientists, there is still a comprehensive network missing needed for understanding the biological details of HCV and the infection pathway of its proteins. There is a need of a single comprehensive network that encompasses the multiple researches done on Hepatitis C virus interactome.

The goal of this study is to get an explicit idea and understanding of hepatitis c viral protein-protein interactions by integrating both large-scale and small-scale studies either conducted experimentally or computationally on HCV interactome. In this way, we will get vast information regarding Hepatitis C virus interactions from both experimentally-determined and computationally-predicted associations. The aim of creating such a comprehensive network is to combine all the previous work done on HCV-Human interactions; to bring forward a new approach to study pathogens and their connections with host organism and to find potential molecules that are involved in causing the disease which can then be knocked down by designing potential drugs. Every disease in a human body is triggered not by a single element but by the mutual interaction of different molecules.

The objective of this study was to find the molecules which were highly involved in stimulating HCV and we came up with certain human genes which were highly interacting with HCV partners but were also actively contributing in other disease and hormonal pathways.

## Methods

The first step of the study was to collect data of all the work done on HCV interactome till date and for this purpose, the PubMed advanced search option was used using multiple keywords related to HCV protein-protein interactions. The search yielded 729 studies containing a variety of literature related the diseases caused by HCV, its epidemiology, gene expression data, and other aspects. The focus of our research was to get protein-protein interactions of HCV with its host *Homo sapiens* and its own proteins. So, we refined the search research results and narrowed down it to 13 studies which exclusively contained PPI data of HCV-Human and HCV-HCV. All the data collected from these research studies was merged and after removing duplicates, a total of 1325 interactions were found among which 21 were HCV-HCV interactions, and 1304 HCV-Human interactions. Table 1 lists the selected studies with their respective number of interactions accessed.

**Table 1:** List of 13 papers with accessible interaction data

S/No.	Paper	No. of Interactions	References
1.	Saik, 2015	907	[17]
2.	David, 2014	1	[18]
3.	Dolan, 2014	6	[19]
4.	Meistermann, 2013	10	[20]
5.	Hagen, 2014	20	[21]
6.	Mukhopadhyay, 2013	549	[22]
7.	Germain, 2013	100	[23]
8.	Dolan, 2013	112	[24]
9.	Tripathi, 2012	132	[25]
10.	Kwofie, 2011	621	[26]
11.	Tripathi, 2010	56	[16]
12.	Roohvand, 2008	4	[15]
13.	De Chassey, 2008	481	[7]

The data used in different studies is not always in the same format; some studies used protein and gene names as identifier, while others used Uniprot or some other identifiers. For network construction in Cytoscape, every protein in the input file must have same identifier throughout, so we used the Uniprot ID Mapping (http://www.uniprot.org/mapping/), an online tool to convert different identifiers into a same required identifier, which in our case was the gene name.

After having a clean and consistent interaction data, we constructed its network using Cytoscape (v3.3.). Cytoscape (http://www.cytoscape.org/) is a freely available platform for network visualization and analysis. It is a powerful tool to study biomolecular interaction networks and high throughput data can be generated by using this tool. Cytoscape does not only construct networks but also can analyze it by using its built-in functions. The next step after network construction was to explore it for understanding the various aspects of the network. There were a total 12 HCV nodes interacting with 940 human genes. The network was studied carefully by looking at each interactor and finding the potential interactions between the nodes. The interactors were dragged far apart from each other in the Cytoscape and every node was explored by looking at its degree distribution across the network.

To find that the set of crucial gene products that are involved in certain pathways and the biological processes targeted by Hepatitis C virus, an enrichment analysis was done using Enrichr (http://amp.pharm.mssm.edu/Enrichr/) of the top 5, 10 and 20 most connected human genes. Enrichr is an open source online tool to analyze gene sets generated by various genome-wide experiments and contains diverse gene sets along with their biological information for any further experimentation or discovery. The enrichment analysis can identify a group of genes which is over represented in a large set of genes, and whose gene products might be critical in causing a particular disease. The Enrichr allow us to perform several types of analysis including Kyoto Encyclopedia of Genes and Genomics (KEGG) pathway analysis, Gene Ontology (GO) enrichment analysis, Online Mandelian Inheritance In Man (OMIM), and several others. KEGG is a comprehensive knowledgebase of 18 databases for interpretation of biological data including genomes, metagenomes and transcriptomes of several organisms. The results obtain from KEGG pathway analysis are based on a combined score by merging the *P*-value and Z-score of the obtained result. Through GO enrichment analysis, we can determine the three main aspects of a gene set, a) its molecular function b) the cellular component c) the biological process in which they are combinedly involved. The role of genes and gene products of any organism can be described by performing its gene ontology.

## Results

**Fig. 1:**
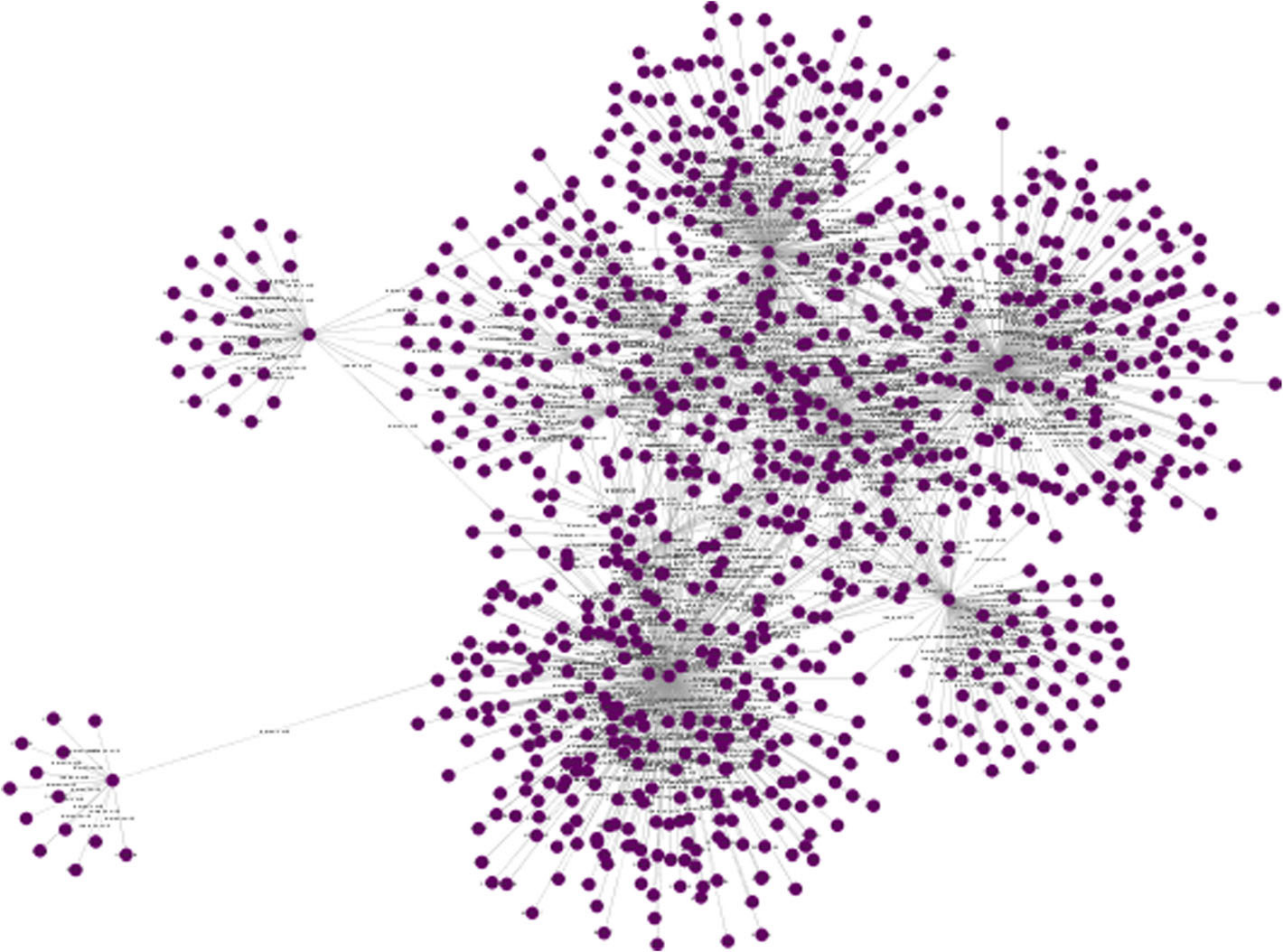
HCV protein interaction network constructed by Cytoscape. The network has 952 nodes in which 12 are HCV proteins and 940 are human genes. There are 1325 edges (interactions) in which 21 are HCV-HCV interactions while 1304 HCV- human protein interactions. The highly connected hubs in the network show HCV proteins interacting with large number of human genes and other HCV proteins. HCV protein interaction network constructed by Cytoscape. The network has 952 nodes in which 12 are HCV proteins and 940 are human genes. There are 1325 edges (interactions) in which 21 are HCV-HCV interactions while 1304 HCV-human protein interactions. The highly connected hubs in the network show HCV proteins interacting with large number of human genes and other HCV proteins

Figure 1 shows the network constructed by Cytoscape in which nodes represents proteins while edges shows the interactions between them. To get a clear idea of network statistics, we used the network analyzer tool in Cytoscape which gives us various useful statistical of the network as shown in Table 2.

**Table 2:** Important statistical measures of HCV-Human network

Attributes	Values
No. of Nodes	952
No. of Edges	1325
Avg. Degree	2.770
High Score Interacting Proteins	NS3, NS5A, Core
Network Density	0.003
Network Diameter	8
Clustering Coefficient	0.078
Shortest Paths	905,352(100%)

**Fig. 2:**
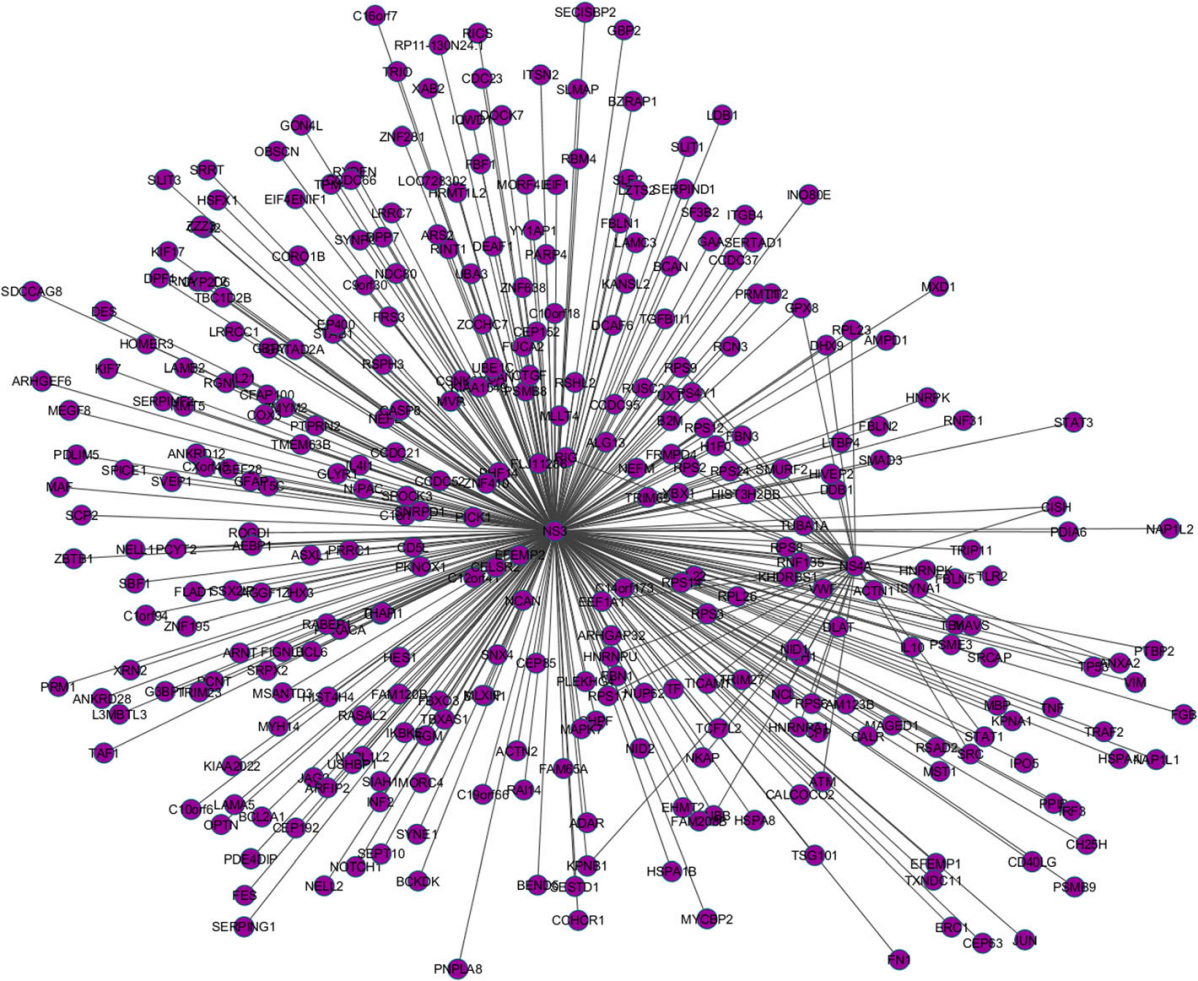
A single cluster within the entire comprehensive HCV-Human protein interaction network. Highly connected nodes interacts with each other and forms a hub. Cytoscape use a number of clustering algorithms including hierarchical, k-means, AutoSOME and k-medoid

**Fig. 3:**
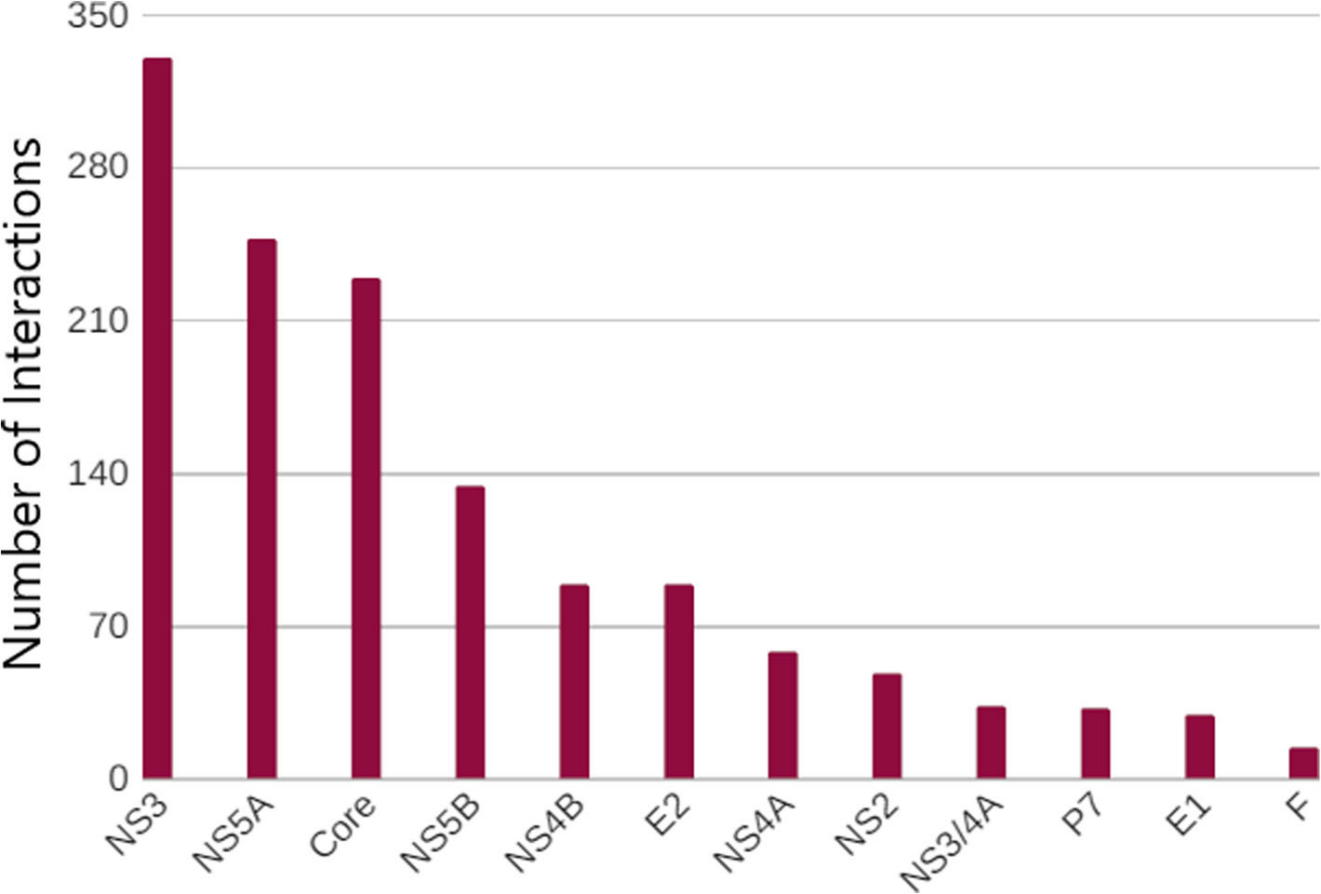
A bar chart representing the distribution of HCV protein interactions

Based on its associations with host genes, HCV proteins were analyzed in the Cytoscape and it was found out that HCV non-structural protein NS3 has the greatest number of interactions with the human genes, i.e., 330 connections. It was followed by NS5A with 247 interactions, and so on. Figure 2 shows the cluster made by the associations of NS3 with host and other viral HCV proteins. The minimum number of interactions an HCV protein had with human genes was 14 by F protein. Figure 3 represents a bar chart of each HCV protein with its relevant number of interactions shown on y-axis while list of top scoring HCV proteins with high percentage of interactions is shown in Table 3.

**Table 3:** List of top scoring interacting proteins

HCV Proteins	No. of Interactions	Percentage
NS3	330	24.7%
NS5A	247	18.5%
Core	229	17%
NS5B	134	10%

By thoroughly exploring and analyzing the network, we found human genes which were interacting with most number of HCV proteins. The highest number of interactions a human gene had with HCV proteins was 6 and the minimum number of interactions an interactor had with HCV proteins was 1. ANXA2 and NR4A1 were interacting with 6 HCV proteins while 11 human genes were interacting with 5 HCV proteins. Likewise, there were common interacting proteins among them too including NS4A, CORE and NS5B which were the common interactors between ANXA2 and NR4A1. The total number of human genes involved in the network was 941, here it should be noted that the enrichment analysis of top HCV connected human genes was performed, i.e., genes with maximum number of associations with HCV proteins. In the current study, top genes were analyzed in Enrichr, upto 20 genes were uploaded to interpret their top-notch functions and to understand their biological significance.

**Fig. 4:**
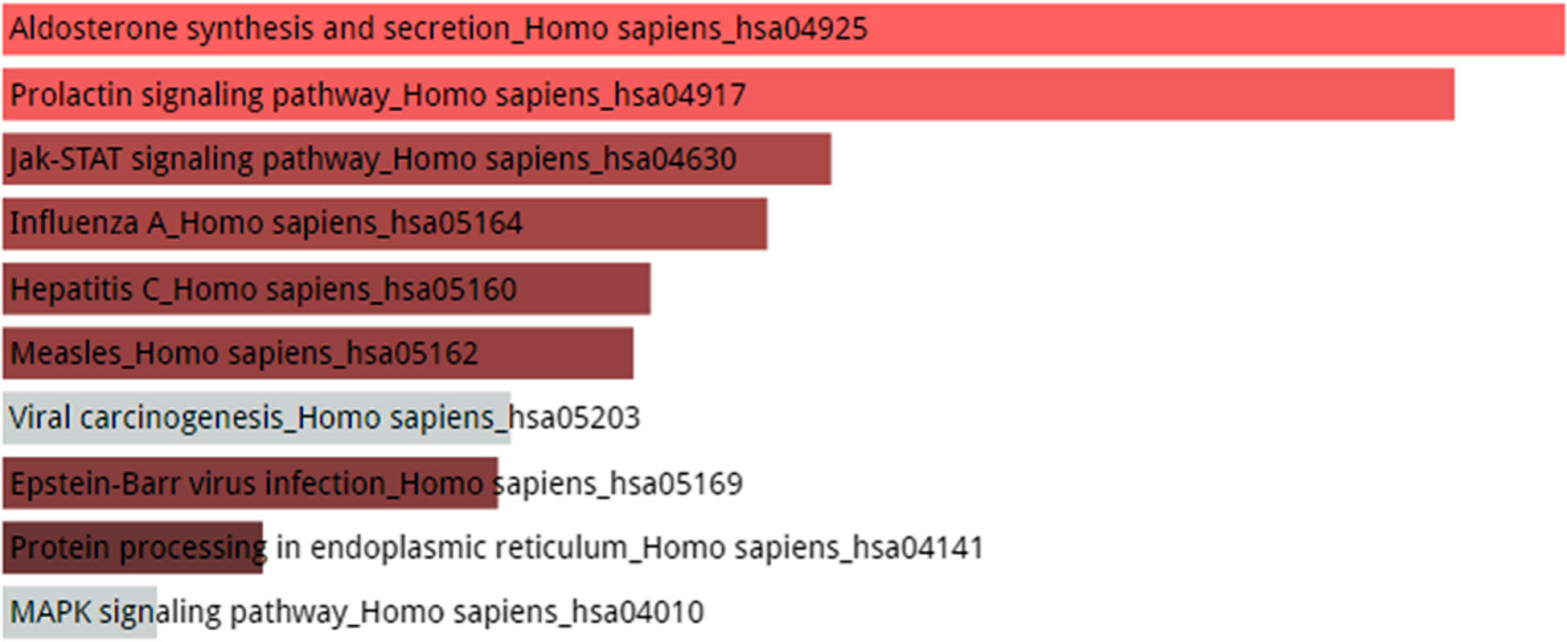
KEGG Pathway enrichment analysis of top 5 most connected genes shows that the genes are enriched in two hormonal pathways, A. Synthesis and secretion of a steroid hormone, the aldosterone and B. The prolactin hormone which is associated in a vast variety of biological functions within human body. Other pathways in which the set of genes were enriched include Jak-STAT signaling pathway, influenza A, hepatitis C, measles, epstein-barr virus infection and protein processing in endoplasmic reticulum. It should be noted here that color of the bars shows the intensity of the enrichment of the specific genes in a certain pathway. The lighter the color, the most enriched the genes are in that specific pathway. In the above picture, pink color bars shows that genes are enriched in only two pathways and the grey color shows that genes are very less or not enriched in the rest of the processes

**Fig. 5:**
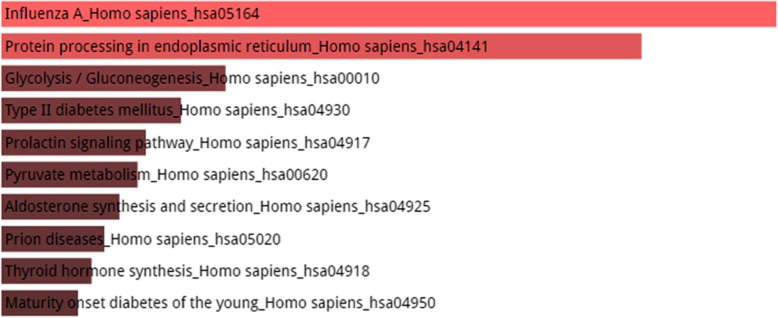
KEGG Pathway enrichment analysis of top 10 genes revealed the fact that combinedly they were actively enriched in human influenza viral infection and the protein processing in endoplasmic reticulum with a score of 6.14. Other pathways include glycolysis, type II diabetes mellitus, prolactin signaling pathway, pyruvate metabolism, aldosterone synthesis and secretion, prion diseases, thyroid hormone synthesis and maturity onset diabetes of the young

**Fig. 6:**
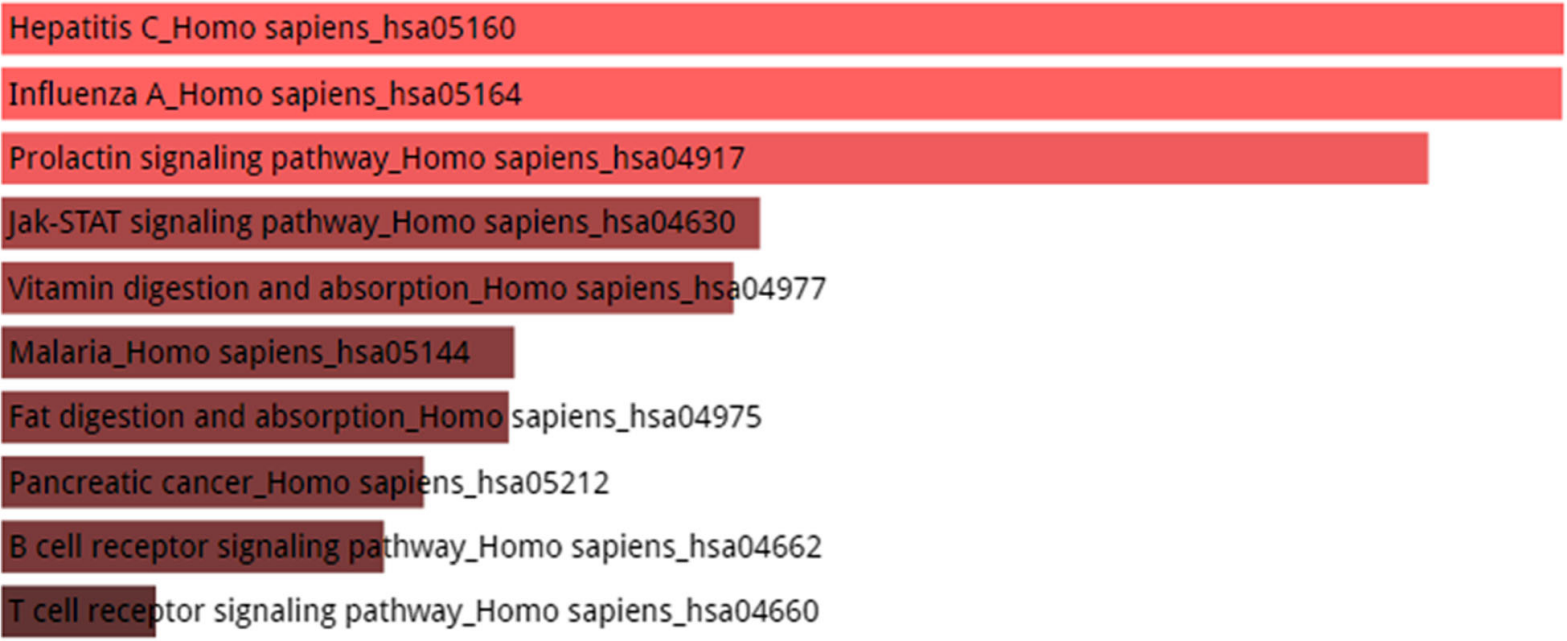
KEGG Pathway enrichment analysis. The set of top 20 genes were enriched in hepatitis C with a score of 12.96 followed by influenza A with a close score of 12.95. Other pathways include prolactin signaling pathway, Jak-STAT signaling pathway, vitamin digestion and absorption, malaria, fat digestion and absorption, pancreatic cancer, B cell receptor signaling pathway and T cell receptor signaling pathway

Figures 4, 5 and 6 shows the KEGG pathway analysis of top 5, 10 and 20 most connected genes respectively. The top 5 most connected genes are highly enriched in two significant hormonal pathways in human body, while KEGG pathway analysis of top 10 most interacting genes revealed that the gene set was involved in human influenza A viral infection and certain other pathways including type II diabetes mellitus. The third KEGG analysis of gene set of top 20 highly interacting genes showed that the genes were highly enriched in Hepatitis C viral pathway and human influenza A viral infection followed by prolactin signaling pathway, Jak-STAT signaling pathway and other biological processes as shown in Fig. 6.

In addition to KEGG pathways analysis, the Gene Ontology (GO) enrichment analysis of the top genes was also performed using Enrichr interface. GO enables us to functionally analyze a set of genes and provides useful information regarding the cellular component, of which the gene set combinedly are part of, the molecular functions of that set of genes and the biological processes in which those genes are actively playing a part.

### MCODE clustering analysis

**Fig. 7:**
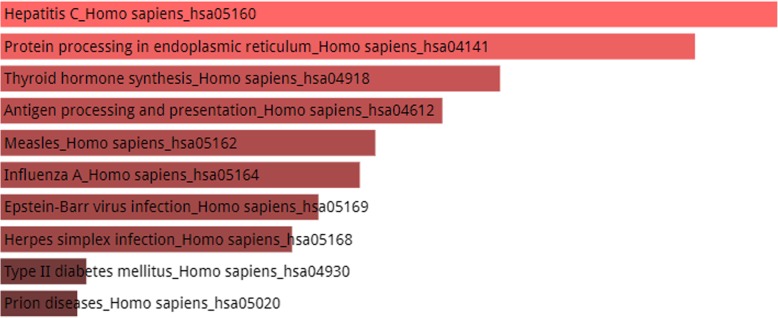
KEGG pathway analysis of the 7 HCV-associated human proteins

We performed MCODE on the whole network and found one cluster in the result. The cluster contained 11 nodes among NS2, Core, E1, NS5B were viral proteins while PKLR, CANX, CD81, HOXD8, EIF2AK2, HSPA5, DDX58 belonged to human. When we performed the KEGG pathway analysis of these human genes, we found out that the genes were collectively involved in Hepatitis C Viral pathway on the top as shown in Fig. 7.

We checked the 7 HCV targets obtained from MCODE clustering analysis on drug repurposing hub (https://clue.io/repurposing-app) and found out that the drug for HCV i.e. ribavirin only targets some human genes which were not in our list of HCV targets.

## Discussion

The results shown in the previous section of the study clearly displays that the analysis of top 5 and top 10 highly interacting manifest that the gene products were more active in certain other pathways rather than hepatitis c viral pathway. This means that the set of proteins were more studied in other disease pathways and are not yet discussed keenly for their role in hepatitis c viral infections. The set of top 10 highly HCV-associated human genes were studied more distinctly in causing influenza A infection. This insight provides us with a clue that the set of genes are highly enriched in HCV but are not well studied in its infection pathway.

The top 5 genes enrichment analysis showed that Hepatitis C is not the only disease in which those genes were actively playing a part. There were other pathways too in which these genes were playing a potential role. ANXA2, DKFZp451G125, NR4A1, CISH and EIF2AK2 were involved in aldosterone synthesis and prolactin signaling pathways more than they were involved in hepatitis c viral pathway.

Previously, another group of scientists (de Chassey et al. 2008) also studied HCV-human interactions experimentally through Y2H and did the KEGG pathway analysis of the human genes interacting with HCV proteins but the difference is that they grouped genes by their interaction with a specific HCV protein and then functionally analyzed their percentage involvement in certain biological pathways. Our study on the other hand, grouped the genes according to their most to least number of associations with hepatitis c viral proteins and then did their KEGG pathway analysis to find out the biological processes targeted by HCV and while using this approach, we found out set of genes which were involved in certain other processes and can be observed as potential targets for scientists working on that specific disease pathway.

## Conclusion

Hepatitis C Virus is the cause of sever and chronic liver diseases including cirrhosis and hepatocellular carcinoma (HCC). Network biology have become the focus of attention in the recent era by scientists for understanding diseases and the biological processes targeted by the disease. My research was centered on constructing an integrated and comprehensive network of hepatitis c virus interactions with its host *Homo sapiens* and then analyzing the network using different techniques. After finding potential targets, we performed the enrichment analysis of the top interacting human genes along with its gene ontologies to find out the biological processes and activities performed by the HCV targeted human genes. In this process of finding potential targets for HCV infection, we found out genes which were involved in other diseases including Influenza A, Hepatitis B and measles. Though the intensity of the genes enriched in the diseases varies from highly enriched to less enriched but still there is a chance of looking for their effects on the disease by switching them on or off in the diseased and normal states. In addition to this, we performed MCODE clustering analysis and found out a group of proteins which were rich in HCV viral pathway but there were no drugs targeting them according to the drug repurposing hub. It can be concluded that the cluster we obtained from MCODE contains potential targets for HCV treatment and could be implemented for molecular docking and drug designing further by the scientists.

## Abbreviations

HCV: Hepatitis C Virus
E1: Envelope glycoprotein 1
E2: Envelope glycoprotein 2
p7: Polyprotein within the HCV polypeptide
NS2: Non-structural protein 2
NS3: Non-structural protein 3
NS4A: Non-structural protein 4A
NS4B: Non-structural protein 4B
NS5A: Non-structural protein 5A
NS5B: Non-structural protein 5B
NS3/4A: complex between non-structural protein 3 and 4A
F: Frameshift protein
ANXA2: annexin A2
NR4A1: nuclear receptor subfamily 4 group A member 1
PPI: Protein-Protein Interaction
Y2H: Yeast 2 hybrid
TAP-MS: Tandem affinity purification combined with mass spectrometry
KEGG: Kyoto Encyclopedia of Genes and Genomics
GO: Gene Ontology
